# Implementing a provisional overarching intervention for COVID-19 monitoring and control in the Brazil-Colombia-Peru frontier

**DOI:** 10.3389/fpubh.2023.1330347

**Published:** 2024-01-08

**Authors:** Matilde Contreras, Felipe Gomes Naveca, Jose Joaquin Carvajal-Cortes, Guilherme F. Faviero, Jorge Saavedra, Eduardo Ruback dos Santos, Valdinete Alves do Nascimento, Victor Costa de Souza, Fernanda Oliveira do Nascimento, Dejanane Silva e Silva, Sérgio Luiz Bessa Luz, Kelly Natalia Romero Vesga, Juan Camilo Grisales Nieto, Vivian I. Avelino-Silva, Adele Schwartz Benzaken

**Affiliations:** ^1^Instituto Leônidas and Maria Deane, Fundação Oswaldo Cruz, Manaus, Brazil; ^2^AHF Global Public Health Institute at the University of Miami, Miami, FL, United States; ^3^AHF Global Public Health Institute, Fort Lauderdale, FL, United States; ^4^Fundação Oswaldo Cruz Ceará, Eusebio, Brazil; ^5^Department of Infectious and Parasitic Diseases, Faculdade de Medicina, Universidade de São Paulo, São Paulo, Brazil; ^6^AIDS Healthcare Foundation, Los Angeles, CA, United States

**Keywords:** COVID-19, SARS-CoV-2, COVID-19 testing, public health surveillance, epidemiological monitoring, indigenous peoples

## Abstract

**Introduction:**

he challenge was to provide comprehensive health resources to a remote and underserved population living in the Brazil-Colombia-Peru border, amid the most disruptive global crisis of the century.

**Methods:**

In August 2021, Fundação Oswaldo Cruz Amazonia (FIOCRUZ Amazônia) and partner collaborators implemented an overarching provisional program for SARS-CoV-2 detection and lineages characterization, training of laboratory personnel and healthcare providers, donation of diagnostic supplies and personal protective equipment, and COVID-19 vaccination. The expedition was conducted at the Port of Tabatinga, a busy terminal with an intense flux of people arriving and departing in boats of all sizes, located in the Amazon River basin. Local government, non-profit organizations, private companies, and other stakeholders supported the intervention.

**Results:**

The expedition was accomplished in a convergence point, where migrant workers, traders, army personnel, people living in urban areas, and people from small villages living in riversides and indigenous territories are in close and frequent contact, with widespread cross-border movement. Using a boat as a provisional lab and storage facility, the intervention provided clinical and laboratory monitoring for 891 participants; vaccination for 536 individuals; personal protective equipment for 200 healthcare providers; diagnostic supplies for 1,000 COVID-19 rapid tests; training for 42 community health agents on personal protection, rapid test execution, and pulse oximeter management; and hands-on training for four lab technicians on molecular diagnosis.

**Discussion:**

Our experience demonstrates that multilateral initiatives can counterweigh the scarcity of health resources in underserved regions. Moreover, provisional programs can have a long-lasting effect if investments are also provided for local capacity building.

## Introduction

### Description of the nature of the problem being addressed and rationale for the proposed innovation

The COVID-19 pandemic has caused an unprecedented burden worldwide. Besides the massive excess in morbidity and mortality directly associated with the dissemination of SARS-CoV-2 (1), the pandemic has also impaired healthcare provision for other conditions (2, 3) and compromised multiple sectors of human activities including education, mobility, recreation, agriculture, economy, among others (4). Notably, the overall impact of COVID-19 has been disproportionate across different regions and populations. Several studies have shown a higher impact of the pandemic in vulnerable populations including black, Hispanic (5), indigenous and migrant communities (6, 7). Factors such as barriers to access health services, pre-existing socioeconomic issues, comorbidities, and a general scarcity of policies that accommodate cultural and contextual specificities of these populations are the key contributors to inequities observed in COVID-19.

In Brazil, several studies have documented the extensive impact of COVID-19 in the Amazon region, including a massive outbreak in Manaus, the capital of Amazonas State, in late 2020 and early 2021. In this dramatic episode, healthcare services collapsed, facilities ran out of hospital beds and oxygen supplies, and providers faced extraordinary difficulties while burdened by logistical challenges and fear (8, 9). Other studies have shown that rural communities living in indigenous territories in the Amazon have been disproportionately impacted by the COVID-19 pandemic (10–12).

Since SARS-CoV-2 transmission occurs essentially through respiratory droplets during person-to-person contact, human mobility and patterns of interpersonal interactions are crucial determinants of COVID-19 dynamics (13). Convergence points, where several people originating from distant regions are in close and frequent contact, are potential hot spots for the dissemination of infectious agents. Hence, these locations are relevant targets for monitoring, and should be prioritized for the implementation of prevention strategies to control the dissemination of SARS-CoV-2, as well as other infectious agents, to vulnerable communities.

The Amazonian Brazil-Colombia-Peru border is a relevant convergence point in South America. This remote region brings together migrant workers, traders, army personnel, people living in urban areas, and people from small local villages living in riversides and indigenous territories, with widespread cross-border movement. It is also considered an exchange point for illegal drugs and other contraband, with limited governmental oversight and precarious health services. Hence, this region may operate as a melting pot, facilitating the transmission of infectious agents that will subsequently spread to distant communities facing even higher vulnerability (14, 15).

This manuscript describes the experience and findings of an overarching provisional program for SARS-CoV-2 detection and lineages characterization, training of laboratory personnel and healthcare providers, donation of supplies, and COVID-19 vaccination implemented at the Brazil-Colombia-Peru border.

## Context

### Setting and population in which the intervention occurs

**Figure 1:**
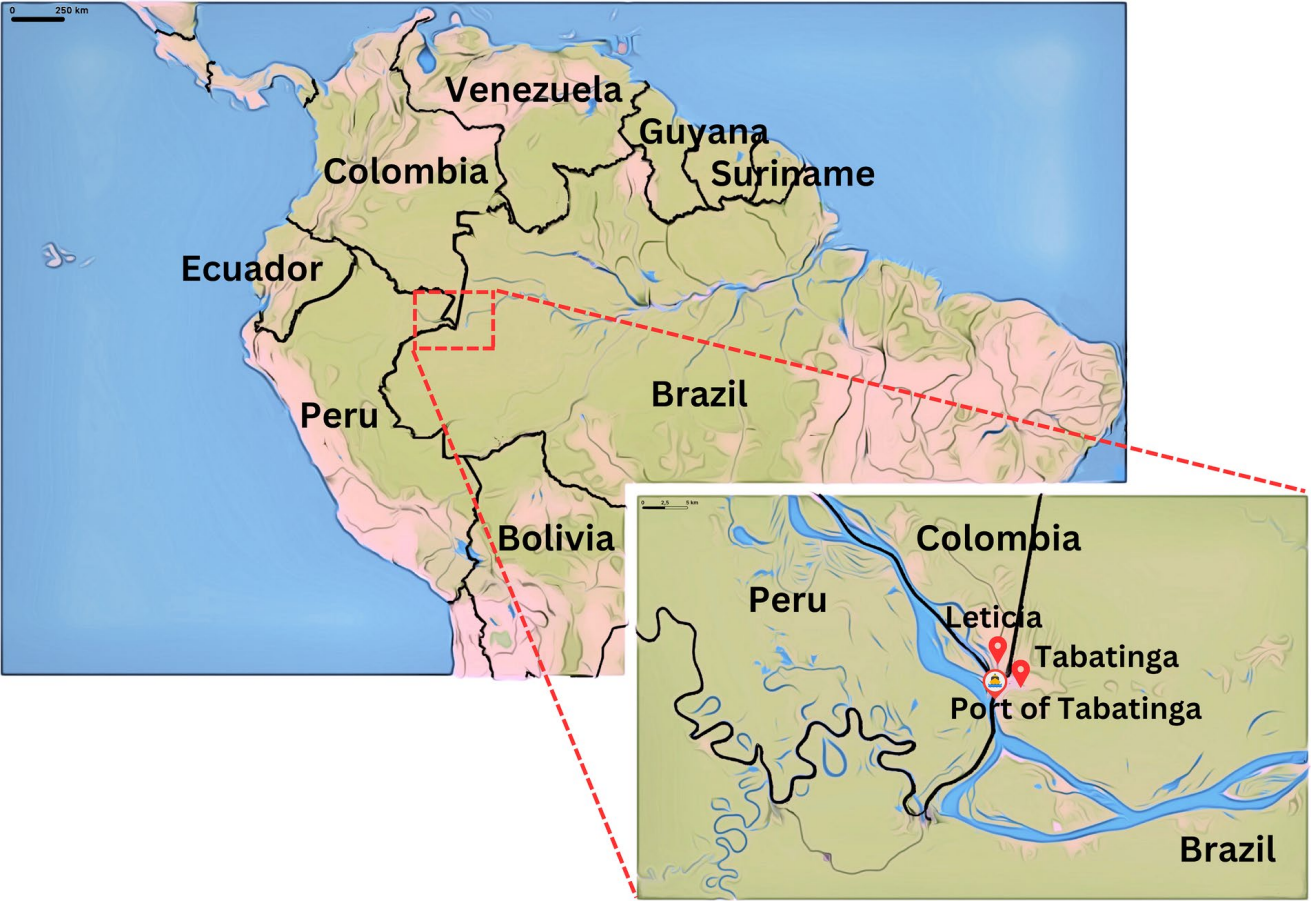
Map of Brazil-Colombia-Peru border and location of the Tabatinga Port.

The Brazil-Colombia-Peru border is a sparsely populated region located in the Amazon River basin, in the microregion of Alto Solimoes. Approximately half of its population live in rural areas, mostly in small villages in riversides and in indigenous territories. The city of Tabatinga is the largest urban center within the Brazilian border, with approximately 67,000 inhabitants (16); its limits are in conurbation with the Colombian city of Leticia, allowing intense cross-border movement of vehicles, people, and goods (17) (Figure 1). The international airport in Tabatinga serves commercial flights and is considered a strategic point for military actions. Finally, the Port of Tabatinga, where our intervention was implemented, is a busy terminal with an intense flux of people arriving and departing in boats of all sizes.

As observed in many regions in the North of Brazil, Tabatinga faces numerous social and healthcare challenges, with limited economic development, low education rates, restricted access to drinking water and sanitation services, and scarcity of health resources including basic infrastructure and supplies, trained human workforce, and diagnostic capacity (18).

Instituto Leonidas and Maria Deane—Fundação Oswaldo Cruz Amazonia (FIOCRUZ Amazônia) is one of most prominent local academic institutions, with vast experience in conducting field activities in remote areas, and solid collaborations established with several local health departments and stakeholders.

The generalized scarcity of health resources in the Brazil-Colombia-Peru border and the complexity of the COVID-19 pandemic underlined the need for an overarching intervention with four main aims: 1. to monitor the rates of prior disease exposure, the local prevalence of active disease, and to identify SARS-CoV-2 variants; 2. to provide training, basic supplies and personal protective equipment for primary care providers; 3. to promote the development of local diagnostic capacity; and 4. to expand COVID-19 vaccination coverage. In short, the expedition’s ambitious goal was to provide overarching health resources to a remote and underserved population in the middle of the most disruptive global crisis of the century.

### Details of key programmatic elements

#### Strategies, players, and partners

To make this bold program become a reality, Fiocruz Amazonia summoned a taskforce that included four researchers, five postgraduate students, four lab technicians, three administrative personnel, nine healthcare providers, and eight volunteers. Tabatinga Health Department was a key collaborator, helping to identify a strategic location and providing street cleaning and police escort for the intervention. Companies in the private sector and nonprofit organizations provided financial resources. The team of providers who had direct contact with participants in the intervention included Portuguese and Spanish-speaking personnel.

#### Setting and procedures

The main setup for the monitoring intervention was organized in a sheltered and ventilated area in a central spot of Tabatinga Port. Professionals dressed in identified vests invited any individuals who were passing by to participate in the intervention. All participants signed an informed consent prior to inclusion. Participants responded to a questionnaire including demographics, prior COVID-19 diagnosis, history of vaccination, contact with COVID-19 suspect or confirmed cases, other risk factors, and presence/duration of COVID-19 symptoms. Next, symptomatic and asymptomatic participants underwent a collection of nasopharyngeal swabs for SARS-CoV-2 rapid antigen tests and real-time polymerase chain reaction (RT-PCR); optionally, a blood sample was collected to quantify antibodies against SARS-CoV-2. Finally, we offered eligible participants the COVID-19 vaccine, using the vaccine platform adopted as a single dose at the time (Janssen). Participants received orientations concerning COVID-19 transmission, prevention, symptoms, and warning signs.

The intervention was approved by the ethics committee at Fundação de Medicina Tropical “Doutor Heitor Vieira Dourado” (approval number 4.879.20).

#### Supporting infrastructure: boat-based lab, storage facility, and housing

Significant challenges for health interventions in the Amazon region include the constant heat, humidity, scarcity of stable refrigeration equipment, mobility constraints, and paucity of nearby laboratory facilities. To overcome these issues in the specific context of an intervention conducted in a port, we opted for using a provisional boat-based lab and storage facility. A rented commercial boat was adapted to store personal protective equipment, rapid tests, and other supplies; to accommodate liquid nitrogen tanks; and to provide housing for part of the team during the intervention. The boat also transported a portable thermocycler for RT-PCR assays that was used during the intervention.

#### Laboratory methods and data capture

Trained personnel performed rapid tests for SARS-CoV-2 using the DPP^®^ SARS-CoV-2 Antigen System (CHEMBIO Diagnostics Brazil) according to the manufacturer’s instructions. Test results were informed with privacy to each participant approximately 25 min after collection. Participants with positive results received additional prevention orientations and a kit with facial masks and alcohol-based hand sanitizer.

SARS-CoV-2 RT-PCR was performed in nasal swab samples using a commercial assay (SARS-CoV-2-E/RP, Biomanguinhos). Samples with a cycle threshold lower than 30 were eligible for sequencing using Illumina COVIDSEQ. Samples for genomic analysis were shipped in liquid nitrogen to Manaus, where sequencing was conducted under the SARS-CoV-2 surveillance program of the Brazilian Ministry of Health, under the auspice of Fiocruz Genomics network.

For serological tests, frozen serum samples were transferred to the central facilities at FIOCRUZ Amazônia and processed using the Human SARS-CoV-2 Spike (trimer) Ig Total ELISA Kit (Invitrogen, BMS2323) and Human SARS-CoV-2 Spike (trimer) IgG ELISA Kit (Invitrogen, BMS2325) following the manufacturer’s instructions. An aliquot was shipped to the COVID-19 Diagnosis Support Unit of FIOCRUZ Ceara and analyzed with SARS-CoV-2 IgG II Quant and SARS-CoV-2 IgG ARCHITECT assay (Abbott Laboratories, Abbott Park, IL, United States; reference 6S60-32/6R86-22). Samples were also tested with a chemiluminescent immunoassay to detect IgG antibodies against the SARS-CoV-2 Spike protein S1 subunit receptor binding domain and SARS-CoV-2 nucleocapsid protein.

We recorded all study data in a secure web application platform [Research Electronic Data Capture, REDCap (19)].

### Monitoring findings

Between August 11th and 19th, 2021, 891 individuals who were 18 years old or older participated in the intervention and provided formal consent for data collection. Demographic characteristics and epidemiological data concerning COVID-19 are presented in Table 1. Most participants were males (54%) of mixed race/skin color (57%) and residents in Brazil (83%). A large percentage (38%) reported traveling in the past 15 days. Half of the participants had not been previously vaccinated for COVID-19; among vaccinated participants, most had received the inactivated Sinovac or the vector-based AstraZeneca vaccine platforms. Thirty-six percent of the study participants reported having had confirmed or suspected COVID-19 in 2020; of those, more than a third received Ivermectin and/or Hydroxychloroquine as treatment. Current COVID-19 among household members and current COVID-19 symptoms were reported by 16 and 19%, respectively (Table 1).

**Table 1:** Demographics and epidemiological data of participants.

Variable	Distribution
Median age in years (IQR)	38 (29–50)
Gender (%)	
Female Male Other	407 (46) 483 (54) 1 (<1)
Race (%)	
White Black Mixed Asian Brazilian indigenous Other/unknown	192 (22) 60 (7) 507 (57) 8 (1) 79 (9) 45 (5)
Number of residents in household (%)	
Up to four Five to eight More than eight	695 (78) 130 (15) 66 (7)
Country of residency (%)	
Brazil Colombia Peru Other	738 (83) 47 (5) 103 (12) 3 (<1)
Presence of ≥ one comorbidity	149 (17)
Travel in the past 15 days	340 (38)
COVID-19 vaccination status	
Unvaccinated Vaccinated with one dose Vaccinated with two doses Unknown	454 (51) 177 (20) 257 (29) 3 (<1)
Vaccine platform	
Sinovac AstraZeneca Other/unknown	169 (39) 195 (45) 73 (17)
COVID-19 diagnosis in 2020	
Confirmed Suspected None	187 (21) 138 (15) 566 (64)
Medications used by suspected or confirmed COVID-19 in 2020 (%)	
Ivermectin Hydroxychloroquine	93 (29) 21 (6)
Has undergone a COVID-19 test in the past 6 months	151 (17)
Had a positive COVID-19 test in the past 6 months	29 (3)
Current COVID-19 among household members	
Confirmed Suspected None	106 (12) 34 (4) 751 (84)

Current COVID-19 symptoms.

Table 2 presents results from diagnostic tests conducted during the intervention. All 891 participants underwent the point-of-care antigen test; in total, 14 participants (2%) had a positive result. Of those, seven reported acute symptoms. Most participants had positive Anti-Spike antibodies by both chemiluminescent and Elisa assays. Antibodies against nucleocapsid antigen were detected in 22% of participants with available samples. Of six participants who had a positive RT-PCR, five were also positive in the rapid antigen test.

**Table 2:** Results from diagnostic tests conducted during the intervention.

Test	Positive frequency (%)
Rapid test	
All participants Symptomatic participants	14 (2) 7 (4)
RT-PCR	6 (1)
Variant	
P.1.12 (Gamma) P.1 (Gamma) P.1.10 (Gamma) B. 1.621.1 (Mu)	3 1 1 1
Chemiluminescent Assay Anti-Spike IgG	
Positive Negative	781 (92) 70 (8)
Chemiluminescent Assay Anti-NC IgG	
Positive Negative	185 (22) 666 (78)
Elisa SARS-CoV-2 Spike (trimer) IgG	
Positive Negative	695 (93) 52 (7)
Elisa SARS-CoV-2 Spike (trimer) Ig total	
Positive Negative	556 (74) 191 (26)

Our team at Fiocruz Amazonia has been conducting SARS-CoV-2 genomics surveillance since the first reported cases in the Amazonas State (20). Thus, one of our goals was to describe the local dynamics of SARS-CoV-2 variants. We found one sample positive for variant B.1.621, categorized as variant of interest (VOI) Mu by the World Health Organization on the same month (21). This variant circulated in low frequencies in the Brazil (22). Furthermore, we identified three lineages of the Gamma variant of concern (VOC), which emerged in the Amazonas State in 2020 (23, 24), continued evolving in 2021 (25), and was latter substituted by Delta and Omicron VOCs (26), without an increase in the mortality despite the upsurge in COVID-19 cases caused by Omicron (27).

Throughout the expedition, we maintained close communication with Tabatinga Department of Health, with immediate notification of all cases detected during the intervention.

### Vaccination

COVID-19 vaccines became available in Brazil in January 2021, with nationwide distribution in public primary care units and temporary vaccination facilities. The first months of the vaccination campaign prioritized older adults, healthcare providers, and people with comorbidities (28). Vaccine acceptance in Brazil was relatively high compared to other countries (29); however, there were heterogeneities in the speed of vaccine rollout across different states, mostly notable in the first semester of 2021.

During the expeditions, 536 vaccine doses were administered to previously unvaccinated participants, of which 65% were Brazilian residents.

### Provision of supplies for local providers

While many regions across the world faced shortages of personal protective equipment, hand sanitizers, and testing supplies during the pandemic, this issue was even more critical in remote and disadvantaged regions.

Our intervention provided 200 personal protective equipment kits comprising a backpack, disposable masks, gloves, an apron, and a face shield to be distributed to local community health agents, including indigenous health agents working in protected territories. We also provided training on personal protection against respiratory droplets, rapid test execution, and pulse oximeter management to 42 community health agents.

### Development of local diagnostic capacity

Most mild and moderate COVID-19 suspect cases in the Brazil-Colombia-Peru border and several other remote regions of Brazil were left without confirmatory investigation due to the paucity of diagnostic resources, compromising the efficacy of isolation and contact tracing strategies. Laboratory investigation for hospitalized cases was also challenging, often requiring shipment of samples to referent institutions, leading to diagnostic delays.

Our expedition supported local diagnostic capacity using two strategies. First, we donated supplies for 1,000 rapid antigen tests to Tabatinga Department of Health and provided training on how to perform the tests to local technicians and providers. Point-of-care rapid tests were shown to be particularly useful in this context as they require no refrigeration or laboratory infrastructure.

We also conducted a hands-on training on molecular biology diagnostics, including tests for SARS-CoV-2 detection and identification of VOCs, to four laboratory technicians working in the Frontier Laboratory (LAFRON) in Tabatinga. Supplies for RT-PCR tests were also provided. A few months after the program was concluded, the local laboratory was able to fully implement RT-PCR testing for SARS-CoV-2 and other infectious agents.

## Discussion

We describe the implementation and results of an overarching provisional program for COVID-19 monitoring, capacity building, and vaccination implemented in the city of Tabatinga, a convergence point in the Brazil-Colombia-Peru border. Tabatinga was selected for its vulnerability concerning health resources and for being a potential hot spot for SARS-CoV-2 transmission and subsequent dissemination to distant communities. The program combined efforts from experienced academic institutions, government stakeholders, non-profit organizations, private sector, and volunteer individuals. The key elements for the successful completion of the program included the provision of resources based on local needs; multisectoral approach; investment in local capacity building; and preparedness, anticipating specific challenges and finding solutions even before they occurred.

Multiple interventions aiming to mitigate the detrimental impact of the COVID-19 pandemic have been implemented across the globe. Provisional interventions included education programs; direct outpatient and inpatient care in temporary facilities; improvised diagnostic units; and large-scale vaccination initiatives (30, 31). Most have been implemented by academic, public health, and governmental institutions in large urban centers, taking advantage of existing resources and infrastructure. However, COVID-19 has disproportionately affected remote and underserved subgroups (32). The scarcity of programs designed to support the specific needs of these communities further intensifies existing inequities. In addition, programs aiming exclusively to deliver prevention or care services, without a preoccupation with local capacity building, will only transiently benefit these populations. Our program was notable for delivering a comprehensive set of resources to mitigate the impact of COVID-19 in a strategic and underserved area.

While most participants in our study were Brazilian, more than a third reported traveling in the past 15 days, underscoring the mobility of people in this convergence region. Thirty six percent reported having had confirmed or suspected COVID-19 in 2020; of those, more than a third were treated with Ivermectin or Hydroxychloroquine, later shown to be ineffective in preventing COVID-19 outcomes (33). Only half of the participants reported partial or complete COVID-19 vaccination; numbers were somewhat lower than national-level data, which showed 58% of the population with least one dose of COVID-19 vaccine in the same period (34). Although more than 90% of participants who underwent testing had seropositivity for Anti-Spike, only 22% were seropositive for Anti-nucleocapsid antibodies; this percentage was slightly below expected values based on reported prior infection rates and anticipated positivity following vaccination with CoronaVac, the most widely used vaccine platform in Brazil in 2021 (35). Altogether, our results highlight the potential role of this convergence point in the dissemination of SARS-CoV-2 to remote, vulnerable communities.

Boats and ships have been recurrently used as floating medical facilities delivering care to riverside and coastal communities, often operated by non-profit organizations or the military forces (36, 37). Smaller vessels can navigate through narrow rivers offering basic healthcare to distant communities, such as villages in the riversides of the Amazon Basin. Large and equipped vessels can offer higher complexity care to coastal underprivileged communities. These initiatives encourage the adoption of alternative resources to overcome barriers and provide state-of-the-art healthcare to neglected populations.

There are additional reasons for enhancing surveillance and laboratory capacity in locations close to large forests. Recent studies have highlighted that complex biomes such as the Amazon rainforest are sanctuaries of zoonotic infections that can potentially leap to human populations, igniting new epidemics (38–40). The introduction of a microorganism originated from wild animals among human populations has likely occurred in the COVID-19 pandemic (41) and in several recent infectious disease outbreaks (42). While protecting natural resources is essential to prevent epizootic events that increase the probability of spilling infections in human populations, environments where humans and wild animals are in close contact should be prioritized for strategic monitoring (43).

The COVID-19 pandemic has exposed and even intensified social inequities globally. Multilateral initiatives combining efforts from academic institutions, government stakeholders, non-profit organizations, private sector, and volunteer individuals can counterweigh the scarcity of health resources in underserved regions. Moreover, provisional programs can have a long-lasting effect if investments are also provided for local capacity building.

### Acknowledgement of conceptual or methodological constraints

Retrospectively, we acknowledge that a few additional elements could have been included in the program aiming to address different levels of COVID-19 prevention and care more comprehensively. For instance, the public health impact of vaccines is directly related to vaccination coverage in each population. Our intervention has not addressed issues related to barriers in accessing COVID-19 vaccines or issues related to vaccine hesitancy. We provided supplies for primary care providers, mainly for individual use; providers and facilities in secondary and tertiary levels of care have not been targeted by our program. Finally, we supported local diagnostic capacity for SARS-CoV-2 infections, but not for other laboratory assays – including those potentially applied for the investigation of differential diagnosis and complications of severe COVID-19 cases. Provisional programs supporting underprivileged communities should, to the greatest extent possible, aim at addressing the target problem with a thorough, muti-level approach.

## Data availability statement

The raw data supporting the conclusions of this article will be made available by the authors, without undue reservation.

## Ethics statement

The study was approved by the ethics committee at Fundação de Medicina Tropical “Doutor Heitor Vieira Dourado” (approval number 4.879.20). The studies were conducted in accordance with the local legislation and institutional requirements. The participants provided their written informed consent to participate in this study.

## Author contributions

MC: Data curation, Formal analysis, Writing – original draft, Writing – review & editing. FG: Data curation, Formal analysis, Writing – original draft, Writing – review & editing. JC-C: Data curation, Formal analysis, Writing – review & editing. GF: Data curation, Formal analysis, Writing – review & editing. JS: Data curation, Formal analysis, Writing – review & editing. ER: Data curation, Formal analysis, Writing – review & editing. VA: Data curation, Formal analysis, Writing – review & editing. VC: Data curation, Formal analysis, Writing – review & editing. FO: Data curation, Formal analysis, Writing – review & editing. DS: Data curation, Formal analysis, Writing – review & editing. SL: Data curation, Formal analysis, Writing – review & editing. KR: Data curation, Formal analysis, Writing – review & editing. JG: Data curation, Formal analysis, Writing – review & editing. VA-S: Conceptualization, Formal analysis, Writing – original draft, Writing – review & editing. AB: Conceptualization, Data curation, Formal analysis, Writing – review & editing.
