# Corrigendum: Implementing a provisional overarching intervention for COVID-19 monitoring and control in the Brazil-Colombia-Peru frontier

**DOI:** 10.3389/fpubh.2024.1431006

**Published:** 2024-05-20

**Authors:** Matilde Contreras, Felipe Gomes Naveca, Jose Joaquin Carvajal-Cortes, Guilherme F. Faviero, Jorge Saavedra, Eduardo Ruback dos Santos, Valdinete Alves do Nascimento, Victor Costa de Souza, Fernanda Oliveira do Nascimento, Dejanane Silva e Silva, Sérgio Luiz Bessa Luz, Kelly Natalia Romero Vesga, Juan Camilo Grisales Nieto, Vivian I. Avelino-Silva, Adele Schwartz Benzaken

**Affiliations:** ^1^Instituto Leônidas and Maria Deane, Fundação Oswaldo Cruz, Manaus, Brazil; ^2^AHF Global Public Health Institute at the University of Miami, Miami, FL, United States; ^3^AHF Global Public Health Institute, Fort Lauderdale, FL, United States; ^4^Fundação Oswaldo Cruz Ceará, Eusebio, Brazil; ^5^Department of Infectious and Parasitic Diseases, Faculdade de Medicina, Universidade de São Paulo, São Paulo, Brazil; ^6^AIDS Healthcare Foundation, Los Angeles, CA, United States

**Keywords:** COVID-19, SARS-CoV-2, COVID-19 testing, public health surveillance, epidemiological monitoring, indigenous peoples

In the published article, an author name was incorrectly written as Dejane Silva e Silva. The correct spelling is Dejanane Silva e Silva.

The authors apologize for this error and state that this does not change the scientific conclusions of the article in any way. The original article has been updated.

